# Targeting oral cancer stemness and chemoresistance by isoliquiritigenin-mediated GRP78 regulation

**DOI:** 10.18632/oncotarget.21338

**Published:** 2017-09-28

**Authors:** Fang-Wei Hu, Cheng-Chia Yu, Pei-Ling Hsieh, Yi-Wen Liao, Ming-Yi Lu, Pei-Ming Chu

**Affiliations:** ^1^ School of Dentistry, Chung Shan Medical University, Taichung, Taiwan; ^2^ Department of Dentistry, Chung Shan Medical University Hospital, Taichung, Taiwan; ^3^ Institute of Oral Sciences, Chung Shan Medical University, Taichung, Taiwan; ^4^ Department of Anatomy and Graduate Institute of Biomedical Sciences, School of Medicine, China Medical University, Taichung, Taiwan

**Keywords:** oral squamous cell carcinomas, isoliquiritigenin, cancer stemness, GRP78, chemoresistance

## Abstract

Cancer stem cells (CSCs) are cells that drive tumorigenesis, contributing to metastasis and cancer recurrence as well as resistance to chemotherapy of oral squamous cell carcinomas (OSCC). Therefore, approaches to target CSCs become the subject of intense research for cancer therapy. In this study, we demonstrated that isoliquiritigenin, a chalcone-type flavonoid isolated from licorice root, exhibited more toxicity in oral cancer stem cells (OSCC-CSCs) compared to normal cells. Treatment of isoliquiritigenin not only inhibited the self-renewal ability but also reduced the expression of CSC markers, including the ALDH1 and CD44. In addition, the capacities of OSCC-CSCs to invade, metastasize and grow into a colony were suppressed by isoliquiritigenin. Most importantly, we showed that isoliquiritigenin potentiated chemotherapy along with downregulated expression of an ABC transporter that is associated with drug resistance, ABCG2. Moreover, a combination of isoliquiritigenin and Cisplatin significantly repressed the invasion and colony formation abilities of OSCC-CSCs. Our results suggested that administration of isoliquiritigenin reduced the protein expression of mRNA and membrane GRP78, a critical mediator of tumor biology. Overexpression of GRP78 reversed the inhibitory effect of isoliquiritigenin on OSCC-CSCs. Furthermore, isoliquiritigenin retarded the tumor growth in nude mice bearing OSCC xenografts. Taken together, these findings showed that isoliquiritigenin is an effective natural compound that can serve as an adjunct to chemotherapy for OSCC.

## INTRODUCTION

Head and neck squamous cell carcinoma (HNSCC) is one of the most prevalent and aggressive malignancies worldwide with a dismal prognosis. The majority of HNSCC is oral squamous cell carcinoma (OSCC) that arises from the mucosal surfaces of the oral cavity, lateral border of the tongue, and floor of the mouth. Over the past few years, the survival rate is still far from satisfactory despite continuous efforts to develop effective treatments [1]. Moreover, recurrence of cancer after chemotherapy remains a major problem that needs to be solved [2]. A recent study has revealed that 3-year overall survival rate of advanced OSCC patients with recurrent tumors was less than 30% [3]. Hence, it is imperative to identify targets and develop novel therapeutic strategies for OSCC.

Resistance to radio/ chemotherapy has been attributed to cancer stem cells (CSCs) [4], which possess tumor-initiating, self-renewal properties and give rise to nontumorigenic progeny [5]. It has been suggested that CSCs play a pivotal role in the relapse, metastasis and therapeutic refractoriness of tumors [6]. Various studies have shown that implementation of treatments against CSCs suppress chemoresistance [7, 8] and increase the effectiveness of conventional therapy [9] across a spectrum of malignancies. Thus, CSC-focused therapy seems destined to be the core of effective anticancer approaches.

Isoliquiritigenin (ISL), a flavonoid from licorice, has been found to exhibit numerous pharmaceutical capacities, such as spasmogenic [10], spasmolytic [10-14], analgesic [12] and anti-inflammatory [15] properties. In addition, accumulating evidence has demonstrated the significant anti-tumor ability of ISL. It has been shown to inhibit angiogenesis via VEGF/VEGFR-2 pathway [16] and induce apoptosis through suppression of COX-2 [17], increase in cyclin-dependent kinase 2 activity [18], or generation of reactive oxygen species [19] in cancer cells. Moreover, it has been found to suppress endometrial [20] or lung [21] tumor growth *in vivo*. In HNSCC, ISL has been demonstrated to inhibit tongue squamous carcinoma cells via the anti-oxidant mechanism [22]. Our previous work also has shown that ISL induces cell cycle arrest, apoptosis, and DNA damage in OSCC cells [23]. Moreover, the DNA repair-associated ataxia telangiectasia mutated (ATM) and phospho-ATM were found downregulated. We demonstrated that low dose of ISL suppressed the malignant phenotypes of OSCCs *in vitro* and retarded tumor growth *in vivo*. Above all, it has been reported that ISL inhibits breast CSCs [24] and chemosensitizes these CSCs via targeting glucose-regulated protein (GRP-78) [25]. Nevertheless, the anti-CSC effect or chemosensitizing potential of ISL on OSCC remains to be determined.

GRP78/BiP/HSPA5 is a major chaperone in the endoplasmic reticulum (ER), which regulates a number of biological functions including tumor proliferation [26] and metastasis [27] as well as chemo/radioresistance [28]. Although GRP78 is located mainly in the ER, it has been reported to be anchored at the cell membrane [29]. Cell surface GRP78 has been found to promote cell proliferation by ERK1/2, p38 MAPK and PI-3K and cell survival by Akt and NF-kB signaling cascades in prostate cancer cells [30]. It has been shown that ISL exerts chemosensitizing effects via GRP78 as GRP78 is the direct target of ISL in inhibiting β-catenin/ABCG2 signaling [25]. In addition, β-catenin signaling has been found to be the key to maintain self-renewal and tumorigenicity of CSCs in HNSCC [31]. Accordingly, it is crucial to examine whether ISL could inhibit OSCC-CSCs or potentiate chemotherapy via downregulation of GRP78. To this end, we assessed the influence of ISL on stemness features of OSCC-SCSs and then evaluated its effect on improvement of chemotherapy. Subsequently, we examined the expression of GRP78 in OSCC-CSCs and tested whether the stemness characteristics were affected following overexpression of GRP78. The tumor transplantation mouse model was utilized as well to confirm the role of GRP78 *in vivo*.

## RESULTS

### Cytotoxic effects of isoliquiritigenin in oral cells

**Figure 1:**
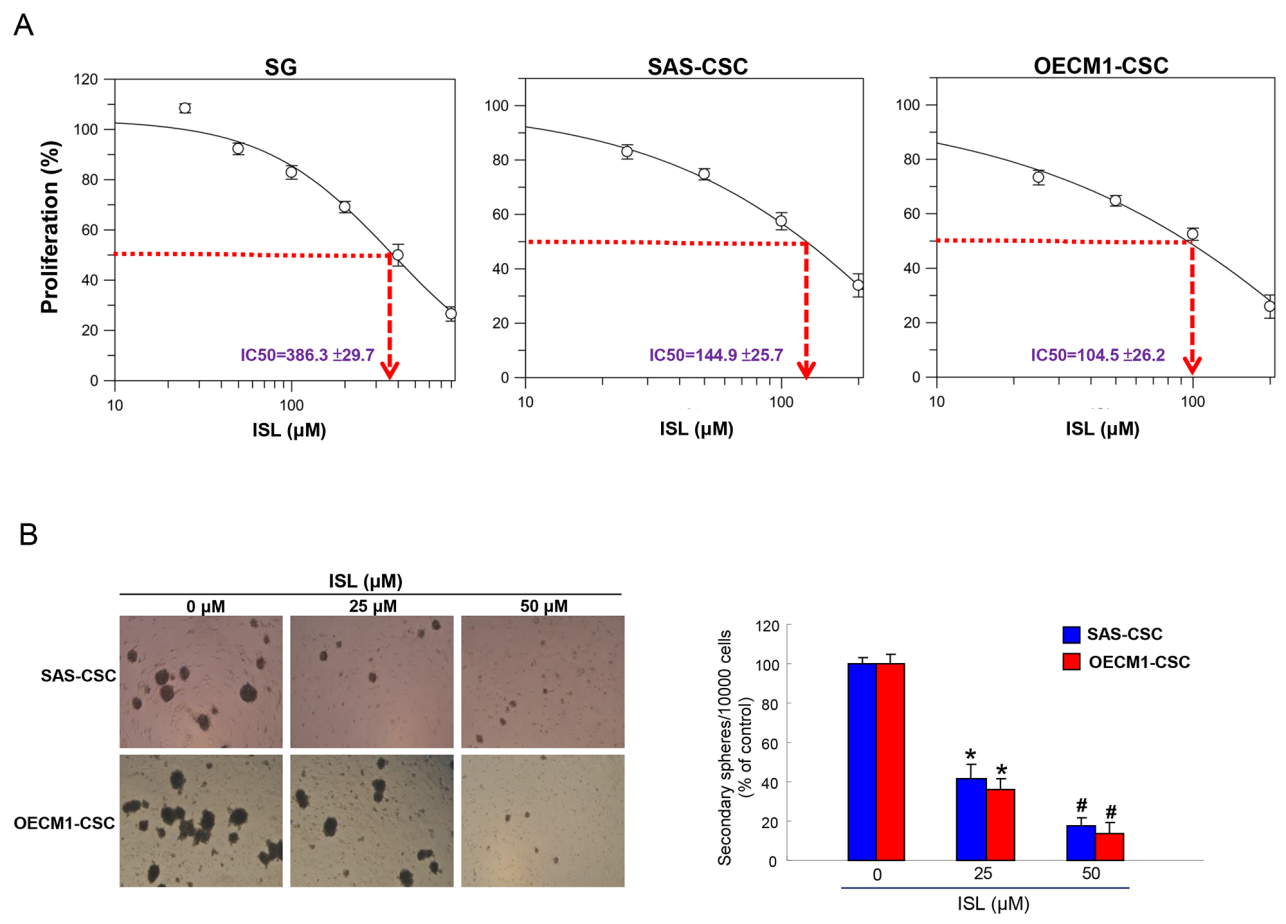
Effects of ISL on cell viability and self-renewal of OSCC-CSCs **(A)** Normal SG cells and OSCC-CSCs derived from SAS and OECM1 cell lines were treated with various concentration of ISL. Cell viability was determined using MTT assay and IC50 values for SG, SAS-CSCs and OECM1-CSCs were 386.3±29.7, 144.9±25.7 and 104.5±26.2 μM, respectively. **(B)** Secondary sphere formation was utilized to assess the self-renewal capacity of OSCC-CSCs with indicated concentration of ISL. The number of the secondary sphere was counted and presented as the percentage control. ^*^p < 0.05 compared to no treatment control group. ^#^*p*< 0.05 compared to 25 μM treatment group or no treatment control group.

We first investigated the impact of ISL on cell viability of an immortalized normal oral epithelial cells (SG) and CSCs-derived from these oral cancer cell lines (SAS and OECM-1) using MTT assay. As shown in Figure 1A, the cytotoxic effect of ISL was more potent in two CSCs than normal SG cells. After treatment with ISL for 24hr, the half maximal effective dose (IC50) of ISL deduced from the generated dose-response curve to SG cells, SAS-CSCs, OECM-1-CSCs were 386.3 ±29.7 μM, 144.9 ±25.7 μM and 104.5 ±26.2 μM, respectively. This finding revealed that the more cytotoxic ISL was to OSCC-CSCs as it required a higher concentration to suppress normal SG cells.

### Self-renewal capacity of CSCs is hindered by addition of isoliquiritigenin

The ability to self-renew is an important feature of CSCs [32] and secondary sphere formation assay has been used widely to evaluate this property [33, 34]. Our results showed that maintenance of self-renewal in these two OSCC-CSCs was inversely affected as the concentration of isoliquiritigenin increased (Figure 1B), suggesting the potential anti-OSCC-CSCs effect of ISL. Consequently, we assessed other stemness signatures in the following experiments.

### Isoliquiritigenin effectively eliminates ALDH1 enzymatic activity and CD44 positivity in OSCC-CSCs

**Figure 2:**
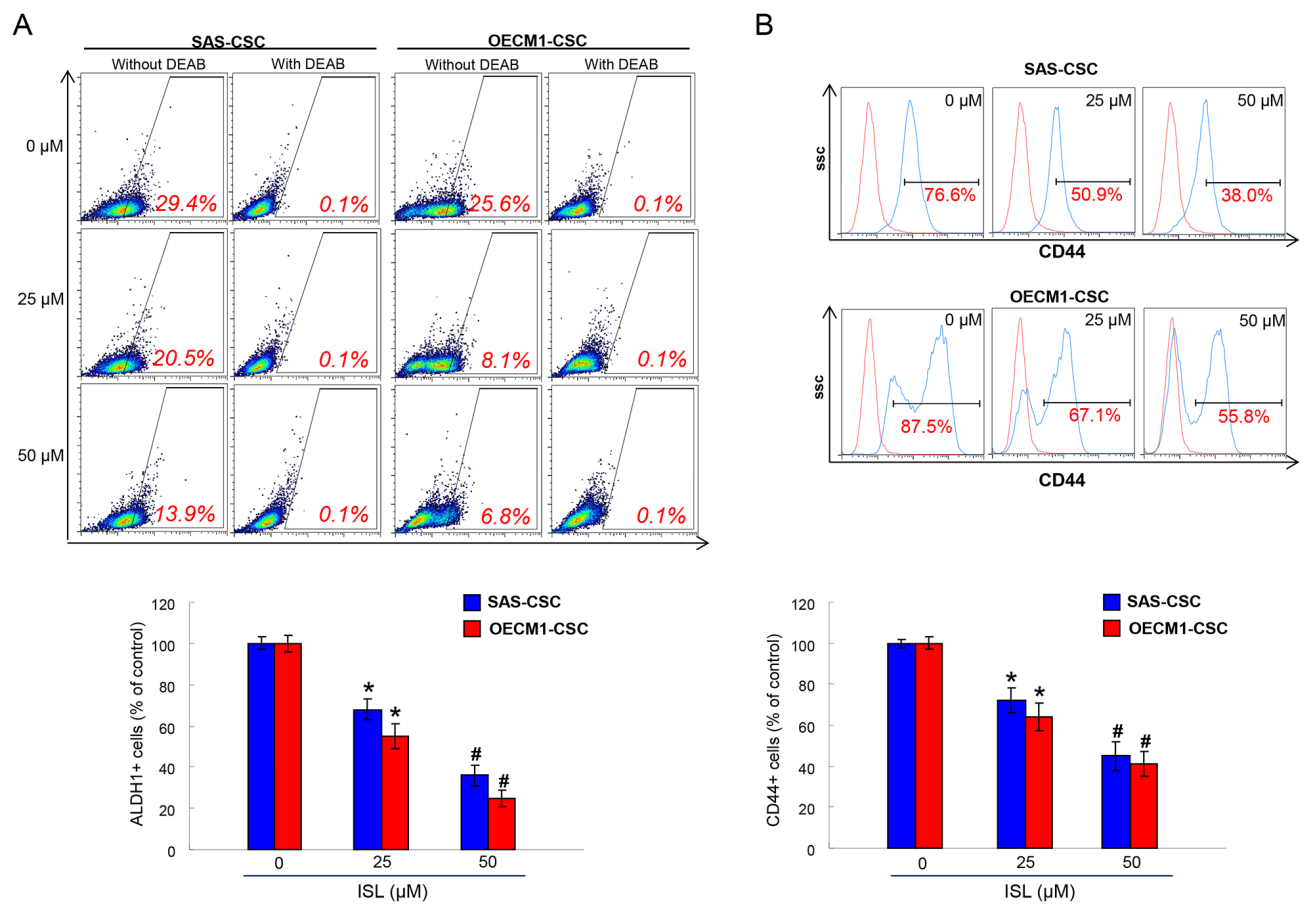
The ALDH1 enzymatic activity and CD44 positivity were reduced with ISL treatment ALDH1 enzymatic activity **(A)** and CD44 positivity **(B)** was gradually eliminated in OSCC-CSCs as the concentration of ISL increased by flow cytometry analysis. Results are means ± SD. ^*^p < 0.05 compared to no treatment control group. ^#^*p*< 0.05 compared to 25 μM treatment group or no treatment control group.

Aldehyde dehydrogenase 1 (ALDH1) is a cytosolic isoenzyme that is responsible for the oxidation of retinol to retinoic acid during early stem cell differentiation and has been proven to be a CSC marker in HNSCC [35, 36]. A commonly used method to identify CSCs is via the Aldefluor Assay (Stemcell Technologies, Inc.) that measures ALDH1 activity with N, N-diethylaminobenzaldehyde (DEAB) as a negative control compound [37]. We observed that ISL induced a concentration-dependent decrease in activity of ALDH1 of both OSCC-CSCs (Figure 2A). Additionally, we examined the expression of CD44, which is also a highly selective marker for CSCs in HNSCC [38]. Flow cytometry analysis of CD44 positivity indicated that ISL-treated OSCC-CSCs expressed a dose-dependently reduction in CD44 expression (Figure 2B). Together, we showed that CSCs markers were gradually down-regulated in both OSCC-CSCs along with the increase in isoliquiritigenin concentration.

### Isoliquiritigenin attenuates the oncogenicity abilities in OCSCs

**Figure 3:**
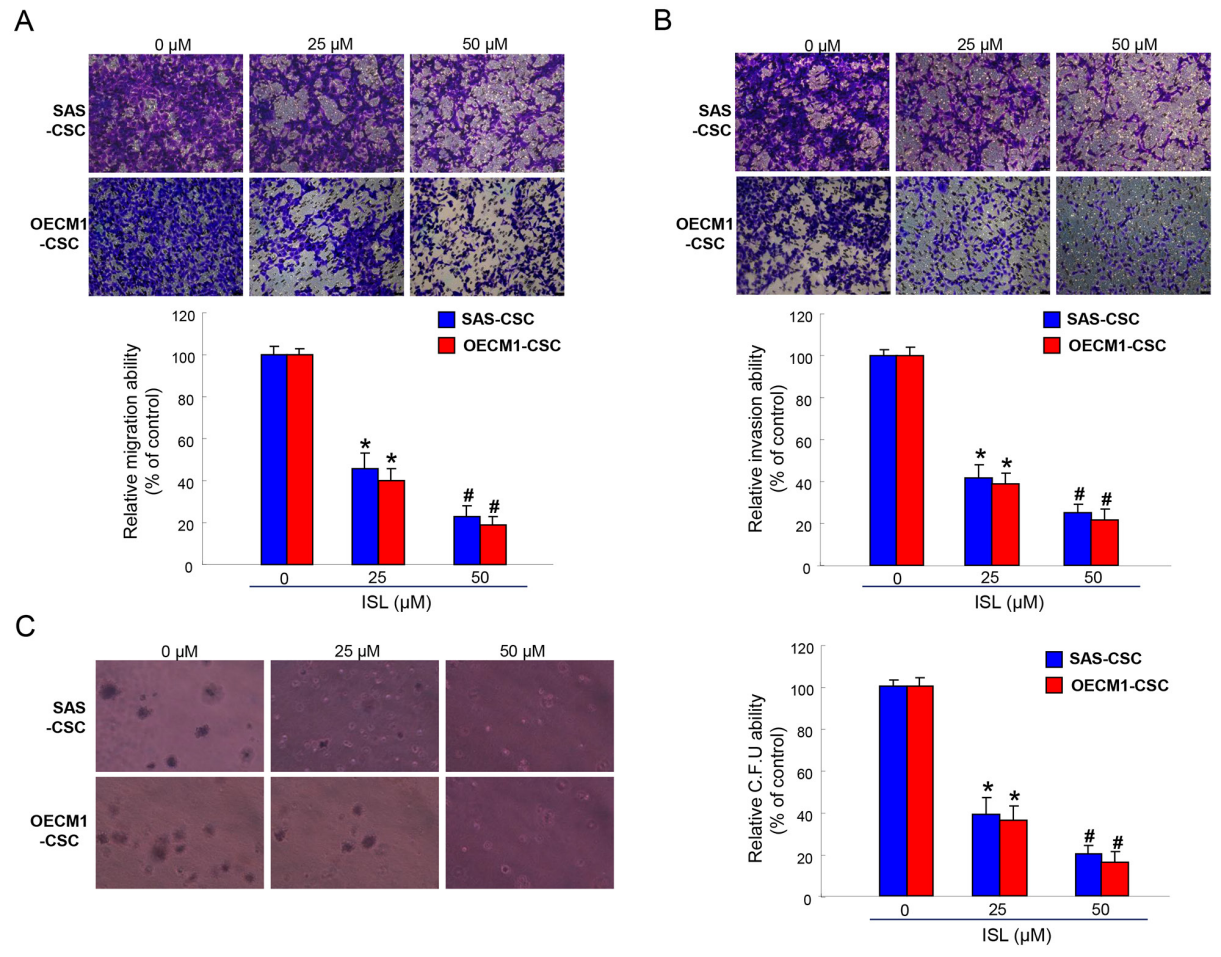
The oncogenicity of OSCC-CSCs was suppressed by ISL Representative images (upper) and quantification (lower) of **(A)** migration assay and **(B)** Matrigel invasion assay of OCSCs treated with various concentration of ISL. **(C)** OCSC with dose-dependent ISL treatment were assigned for the colony formation assay. Experiments were performed in triplicate. Values are expressed as mean ±SD. ^*^p < 0.05 compared to no treatment control group. ^#^*p*< 0.05 compared to 25 μM treatment group or no treatment control group.

Given that CSCs exhibit high tumor-initiating and metastasis capacities and are associated with therapeutic refractoriness of cancers [6, 39], we sought to measure the effects of ISL on colony forming, migration and invasion abilities of OSCC-CSCs. Single-cell suspensions of ISL-treated OSCC-CSCs were used for analysis of their metastatic capacity *in vitro* as described in the Methods section. Our data suggested that ISL dose-dependently suppressed the migration (Figure 3A) and invasion (Figure 3B) abilities of OSCC-CSCs. Anchorage-independent growth assay showed that colony formation capacity of OSCC-CSCs was inhibited by ISL (Figure 3C). These results indicated that ISL significantly suppressed the cologenicity and metastasis potential of OSCC-CSCs.

### Isoliquiritigenin potentiates chemotherapy in the treatment of OSCC

**Figure 4:**
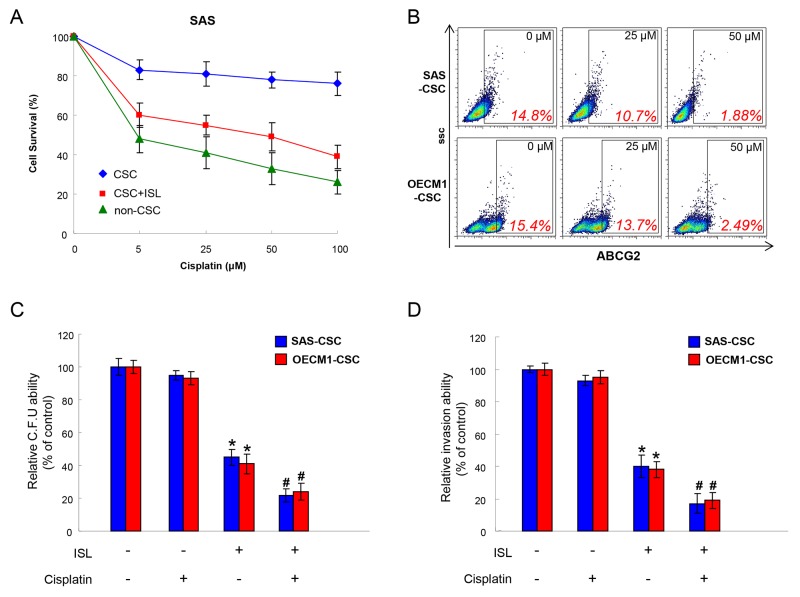
Chemoresistance in OSCC-CSCs is attenuated with ISL treatment **(A)** Cell survival of non-CSCs, CSCs and CSCs+ ISL after Cisplatin were examined using MTT assay. **(B)** The expression of ABCG2 in two OSCC-CSCs was tested by flow cytometry following treated with various concentration of ISL. **(C)** Colony formation and **(D)** invasion capacities after Cisplatin treatment were evaluated with or without ISL. Data were shown as the mean ± SD. ^*^p < 0.05 compared to no treatment control group. ^#^ *p*< 0.05 compared to Cisplatin alone group.

Drug resistance is a crucial cause of treatment failure [40] and it has been attributed to the existence of CSCs [41]. As expected, the cell survival of OSCC-CSCs was barely affected by Cisplatin compared with non-CSCs, while the combination of ISL markedly repressed the number of CSCs (Figure 4A). ABCG2, an ATP-binding cassette transporter protein, is not only a stem cell marker but also associated with drug resistance since ABCG2 has the capacity to transport a broad range of substrates [40-43]. We showed that expression of ABCG2 was downregulated with the increased concentration of ISL (Figure 4B). Thereafter, we assessed the colony formation and invasion capacities to investigate whether ISL could enhance the effect of Cisplatin. Results of these two analyses showed that administration of ISL alone or combined with Cisplatin both reduced the colony forming and invasion abilities of OSCC-CSCs (Figure 4C and 4D). Collectively, we demonstrated that ISL possesses the potential to serve as an adjunct to chemotherapy.

### Anti-CSCs effect of isoliquiritigenin is via downregulation of GRP78

**Figure 5:**
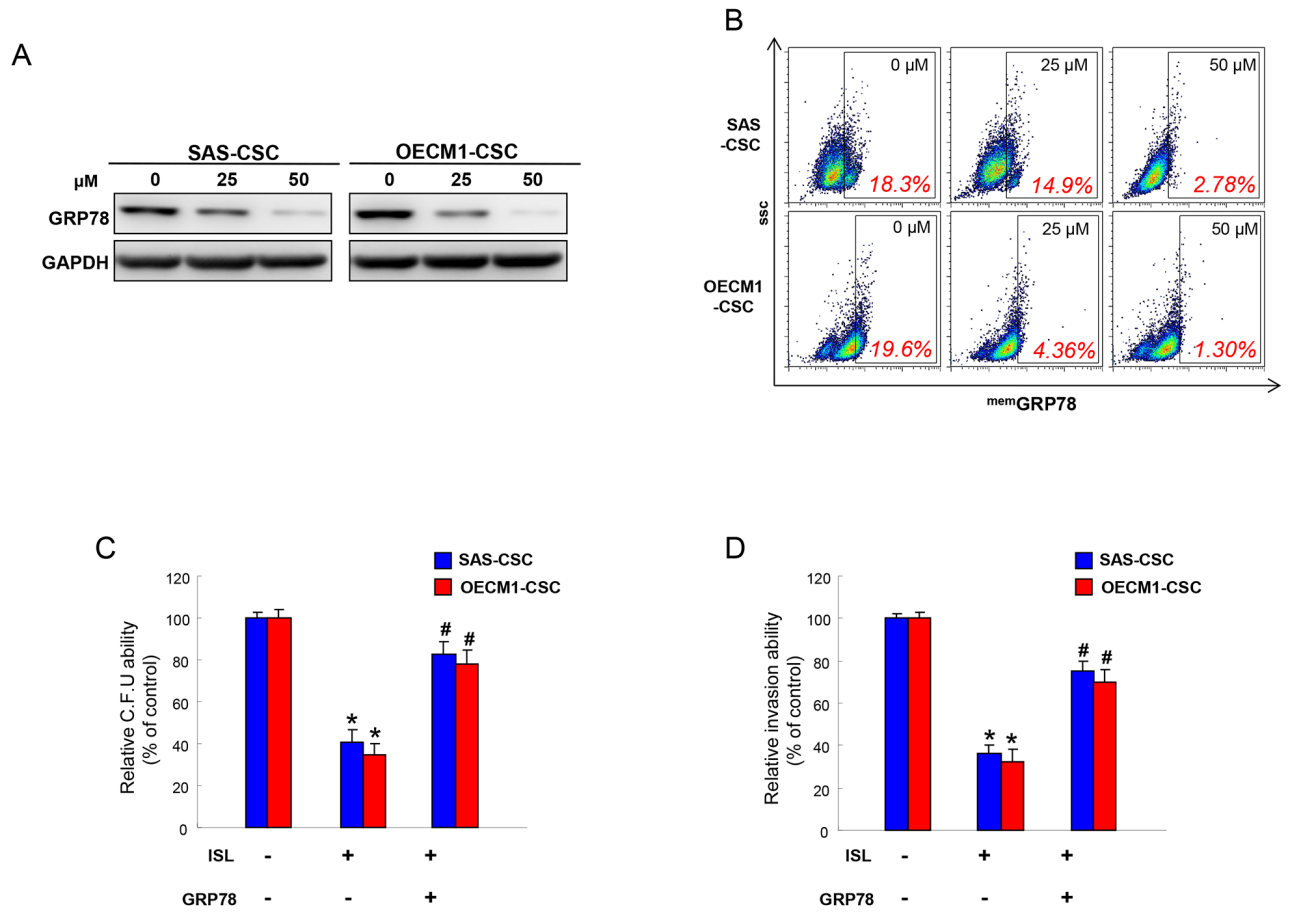
The anti-tumor effect of ISL is mediated by GRP78 **(A)** Protein levels of GRP78 in OCSCs were evaluated in response to ISL treatment by western blotting. **(B)** Membrane-associated GRP78 positive (^mem^GRP78^+^) cells in OSCC-CSCs were analyzed in OCSCs with ISL treatment by flow cytometry analysis. Clonogenicity **(C)** and invasion **(D)** capabilities of OCSCs were examined in the absence or presence of ISL and GRP78. Data were shown as a percentage of no treatment group. ^*^p <.05 compared to no treatment group; ^#^ p<.05 compared to ISL only group.

Our previous work has revealed that the transcripts and protein levels of GRP78 were significantly upregulated in enriched OSCC-CSCs [44]. To elucidate the molecular mechanisms by which to mediate the anti-CSCs effect of ISL, we evaluated the expression of GRP78 after treatment of ISL. Protein expression level of GRP78 was reduced following increased concentration of ISL (Figure 5A). Likewise, membrane-associated GRP78 positive (^mem^GRP78^+^) cells in OSCC-CSCs were also downregulated in response to the addition of ISL by flow cytometry analysis (Figure 5B). Moreover, we found that the overexpression of GRP78 reversed the inhibitory effect of ISL on colony forming (Figure 5C) and invasion (Figure 5D) abilities of OSCC-CSCs. These results suggested that the ISL-inhibited CSCs effect was associated with GRP78.

### Administration of isoliquiritigenin suppressed the tumor growth *in vivo*

**Figure 6:**
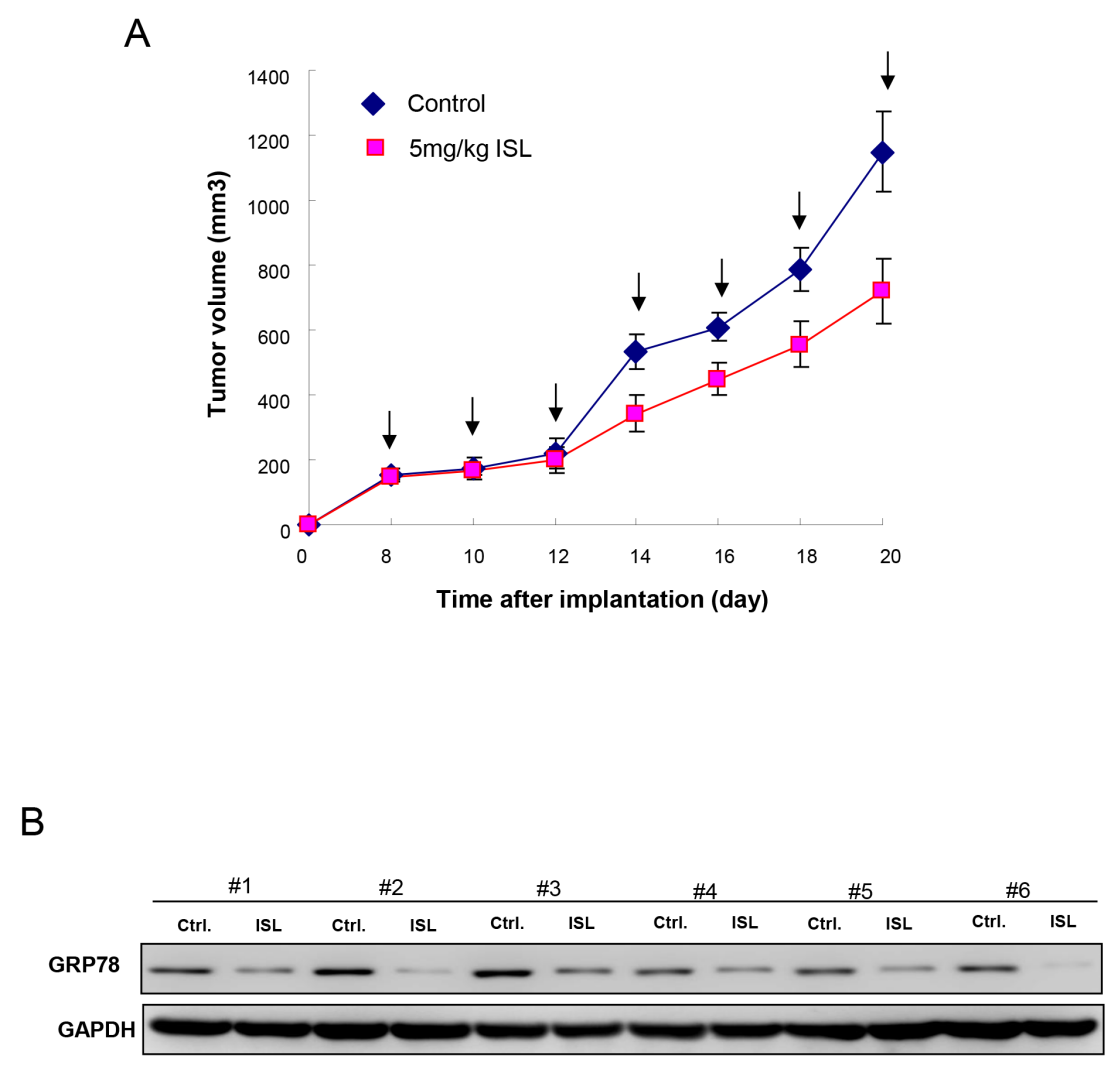
Therapeutic efficiency of ISL in OCSC-transplanted mice **(A)** Tumor size was evaluated in control and ISL treatment (5mg/kg) groups over time until day 20. **(B)** After sacrifice, the expression of GRP78 in the excised tumors from both groups (n=6 for each group) was detected using Western blot. Values are expressed as mean ±SD. ^*^ *p* <.05 compared to control.

Tumorigenesis *in vivo* is a significant aspect when evaluating the anti-CSC effect. Our results showed that oral administration of ISL successfully delayed the tumor growth after transplanting tumor cells into nude mice (Figure 6A). Furthermore, we demonstrated that the expression of GRP 78 was decreased in the tumor tissues extracted from the ISL-treated group by immunoblotting (Figure 6B), suggesting the tumor-suppressive potential of ISL *in vivo*.

## DISCUSSION

Accumulating evidence has attributed the chemotherapy failure or metastasis to the existence of CSCs [45, 46]. In this study, we examined the effect of ISL on the self-renewal, metastatic capacities, and sensitization of chemotherapy of OSCC-CSCs. We found that ISL reduced the expression of CSC markers, the ability of self-renewal and colony formation by reducing the expression of GRP78. Furthermore, our findings suggested ISL enhanced the treatment effect of Cisplatin with reduced CSCs properties and ABCG2 expression. Numerous studies have shown that ABCG2 expression is associated with stemness and drug resistance [40-43], which may lead to treatment failure. The reduced ABCG2 expression following ISL treatment in our study was in accordance with a previous report showing ISL chemosensitizes breast CSCs via GRP78/ β-catenin/ABCG2 signaling.

Several studies have suggested that GRP78 was crucial for enhancement of tumor cell proliferation, protection against apoptosis, and promotion of tumor angiogenesis [47-50]. Our previous study has identified GRP78 as a putative candidate on mediating the stemness hallmarks of OSCC-CSCs by differential systemic analyses. We showed that ^mem^GRP78^+^ OSCC-CSCs displayed stemness properties of self-renewal and radioresistance. In addition, we demonstrated that downregulation of GRP78 inhibited tumorigenicity, whereas overexpression of GRP78 in OSCC-CSCs enhanced the malignant potentials and ^mem^GRP78^+^ expression profile [44]. In the current study, we utilized ISL-mediated downregulation of GRP78 to suppress the oncogenicity of OSCC-CSCs *in vitro* and *in vivo*. In consistent with other studies showing silencing of GRP78 decreases cell growth and sensitizes cancer cells to chemotherapy [51, 52], our results proved that downregulation of GRP78 by administration of ISL also enhanced the treatment effect of Cisplatin (Figure 4). A limitation of the current study was the xenotransplantation model of OSCCs, further studies may utilize carcinogenic agents to induce oral cancer in animals and evaluate the effect of ISL on prevention of OSCCs development.

It has been shown that ISL induced growth inhibition and apoptosis in human breast cancer cells via deactivation of PI3-K/Akt pathway [53] or in endometrial cancer via activation of ERK [20]. In this study, we found that ISL reduced the expression of GRP78, and decreased expression of GRP78 has been found to exert pro-proliferative and anti-apoptotic properties in prostate carcinoma cells through activation of MAPK and PI3-K pathways [30]. Another study has revealed that Cripto/GRP78 complex mediated oncogenic signaling via MAPK, PI3-K and Smad2/ 3 pathways in mammary cells [54]. Likewise, blockage of GRP78 expression suppresses tumorigenesis and Akt activation with PTEN loss in prostate epithelium [55]. As for OSCC-CSCs, we have shown that knockdown of GRP78 induced apoptosis via enhanced expression of PTEN, BAX, and caspase3 as well as reduced expression of p-MAPK in our previous work [44]. Further studies are required to clarify whether PI3-K/ Akt or MAPK pathways are involved in the anti-CSCs effect following ISL-mediated GRP78 downregulation.

In conclusion, we demonstrated that ISL has the potential to serve as an anti-CSCs agent with its inhibitory effect on the CSC characteristics, including the expression of CSC markers, self-renewal, metastasis, colony formation capacities as well as enhancement of chemotherapy. Our results suggested that ISL-mediated reduction of GRP78 in OSCC-CSCs played a critical role in these phenomena. Furthermore, we showed that oral administration of ISL successfully retarded tumor growth in xenotransplanted mice. Altogether, these findings support that ISL may be used as a nutritional intervention for an adjunct to chemotherapy in clinical application.

## MATERIALS AND METHODS

### Reagents and cell lines cultivation

ISL was purchased from Sigma–Aldrich (St. Louis, MO, USA). SG cells (Smulow–Glickman human gingival epithelioid cells) were cultured in Dulbecco modified Eagle’s medium (DMEM) containing 10% fetal bovine serum (FBS). SAS cells (high-grade human tongue carcinoma cell line) were cultured in DMEM supplement with 10% FBS and F-12. OECM-1 cells (oral gingival squamous cell carcinoma cell line) were grown in RPMI supplemented with 10% FBS using previously described protocols [56]. All cells were maintained in an incubator with 5% CO_2_ and 95% humidity at 37°C.

### Cell viability

SG, SAS, or OECM-1 cells were plated in 96-well plates at 1 × 10^4^ cells/well in 0.1% DMSO or various concentration of ISL-containing medium (25, 50, 100, 200 to 400 μM) and cultured at 37°C for 24 h. Cell proliferation/ survival was determined by MTT (3-(4,5-dimethylthiazol-2-yl)-2,5-diphenyl tetrazolium bromide) assay. The 570-nm absorbance of the DMSO-treated group was set as 100% and data were presented as a percentage of DMSO control [57].

### Secondary sphere formation

OSCC cells were dissociated and cultured as tumorspheres in modified DMEM/F-12 supplemented with N2 (R&D), 10 ng/mL epidermal growth factor (EGF, Invitrogen, Carlsbad, CA, USA), 10 ng/mL basic fibroblast growth factor (bFGF, Invitrogen), and penicillin/streptomycin at 10^3^ live cells/low-attachment 6-well plate (Corning, Acton, MA, USA). The medium was changed every other day until the tumor sphere formation was observed in about 2 weeks [58].

### Aldefluor assay and flow cytometry analysis

To measure the ALDH1 activity, Aldefluor assay was performed according to manufacturer’s (Stemcell Technologies, Durham, NC, USA) instruction. Dissociated single cells were suspended in Aldefluor assay buffer containing ALDH substrate, and ALDH inhibitor, diethylaminobenzaldehyde (DEAB), was used as negative control.

Cells were stained with anti-CD44 (Miltenyi Biotech., Auburn, CA, USA), or anti-^mem^GRP78 (Cell Signaling, Danvers, MA, USA) or anti-ABCG2 (Santa Cruz Biotechnology, Santa Cruz, CA, USA) antibodies conjugated to phycoerythrin according to the manufacturer’s instructions. Red (>650 nm) fluorescence emission from 10,000 cells illuminated with blue (488 nm) excitation light was measured with FACSCalibur (Becton Dickinson, Mountain View, CA, USA) using CellQuest software [44, 57].

### Migration/ invasion assays

1 × 10^5^ cells in medium with lower serum (0.5% FBS) or various concentration of ISL were seeded into the top chamber of a transwell (Corning, Acton, MA, USA) with a porous transparent polyethylene terephthalate membrane (8.0 μm pore size). Medium supplemented with higher serum (10% FBS) was used as a chemoattractant in the lower chamber. For invasion assay, the membrane was coated with Matrigel™ (BD Pharmingen, NJ, USA). Cells were incubated for 24 h and cells that did not migrate through the pores were removed by a cotton swab. The migrated cancer cells were then visualized and counted from 5 randomly selected fields under 100-fold magnification using an inverted microscope [59].

### Anchorage-independent growth assay

Each well of a 6-well plate was coated with 2 ml bottom agar (Sigma–Aldrich) mixture (DMEM, 10% (v/v) FCS, 0.6% (w/v) agar). After the bottom layer was solidified, 2 ml top agar-medium mixture (DMEM, 10% (v/v) FCS, 0.3% (w/v) agar) containing 2 × 10^4^ cells and various concentration of ISL was added, and the plates were incubated at 37°C for 4 weeks. Plates were stained with 0.005% Crystal Violet and the number of total colonies with a diameter ≥100 μm was counted over five fields per well for a total of 15 fields in triplicate experiments [60].

### RNA isolation and quantitative real-time reverse-transcriptase (RT)-PCR

RNA was extracted from cells using Trizol reagent (Invitrogen) and reverse-transcribed using the Superscript III first-strand synthesis system (Invitrogen). Amplification was carried out on an ABI StepOne™ Real-Time PCR Systems (Applied Biosystems, Carlsbad, CA, USA) using SYBR green I (Roche Molecular Systems, Alameda, CA, USA). PCR reactions were prepared in duplicate and heated to 95°C for 10 min followed by 40 cycles of denaturation at 95°C for 10 s, annealing at 55°C for 5 s, and extension at 72°C for 20 s. Standard curves (cycle threshold values versus template concentration) were prepared for each target gene and for the endogenous reference (GAPDH) in each sample. All reagents and protocols were from Applied Biosystems, and detection was performed using 7900HT fast real-time PCR system [61].

### Stable overexpression of GRP78 in OSCC-CSCs

The cDNA fragments encoding full-length GRP78 was cloned into the pLV-EF1α-MCS-IRES-GFP vector from Biosettia Inc. (Biosettia, San Diego, CA, USA). Lentivirus production was performed by co-transfection of plasmid DNA mixture with lentivector plus helper plasmids (VSVG and Gag-Pol) into 293T cells (American Type Culture Collection, Manassas, VA, USA) using Lipofectamine 2000 (LF2000, Invitrogen, Carlsbad, CA, USA) as previously described [62]. Stable GRP78-overexpressing OCSCs were further purified by cell sorting with GFP positive cells.

### Bioluminescence imaging measurement of tumor growth *in vivo*

All procedures involving animals were in accordance with the institutional animal welfare guidelines of the Chung Shan Medical University. For the nude mice xenograft model, 5–6 weeks old immunodeficient nude mice (BALB/c nu/nu mice) weighing 18–22 g were used. The mice were housed with a regular 12 h light/12 h dark cycle and ad libitum access to standard rodent chow diet (Laboratory Rodent Diet 5001, LabDiet, St. Louis, MO) and were kept in a pathogen-free environment at the Laboratory Animal Unit. SAS-derived sphere-forming OSCC-CSCs (5 × 10^4^ cells/0.2 mL/mouse) were injected subcutaneously into the right front axilla. Eight days postimplantation, the mice were randomly divided into two groups (N = 6 for each group) and fed by oral gavage with saline (control) and ISL (5mg/day/kg) suspended in saline. The day of cell implantation was designated day 0. Bioluminescence imaging (BLI) was performed using an IVIS50 animal imaging system (Xenogen Corp., Alameda, CA, USA). The photons emitted from the target site penetrated through the mammalian tissue and could be externally detected and quantified using a sensitive light imaging system. The image acquisition time was 1 min. The displayed images of the tumor sites were drawn around and quantified in photons per second using Living Image software (Xenogen Corp.) The volume was calculated (according to the following formula: ([length × width^2^]/2) and then analyzed using Image-Pro Plus software. After 20 days, the animals were euthanized, and the primary tumors were isolated and weighed [57].

### Statistical analysis

SPSS (version 13.0) was used for statistical analysis. Student’s *t* test or ANOVA analysis were used to determine the statistical significance of the differences between experimental groups; p values less than 0.05 were considered statistically significant.
